# The effects of a novel aliphatic-chain hydroxamate derivative WMJ-S-001 in HCT116 colorectal cancer cell death

**DOI:** 10.1038/srep15900

**Published:** 2015-10-29

**Authors:** Yu-Han Huang, Shiu-Wen Huang, Ya-Fen Hsu, George Ou, Wei-Jan Huang, Ming-Jen Hsu

**Affiliations:** 1 Graduate Institute of Medical Sciences, College of Medicine, Taipei Medical University, Taipei, Taiwan; 2 Graduate Institute of Pharmacology, College of Medicine, National Taiwan University, Taipei, Taiwan; 3 Division of General Surgery, Department of Surgery, Landseed Hospital, Taoyuan, Taiwan; 4 Department of Medicine, University of British Columbia, Vancouver, British Columbia, Canada; 5 Graduate Institute of Pharmacognosy, Taipei Medical University, Taipei, Taiwan; 6 Department of Pharmacology, School of Medicine, College of Medicine, Taipei Medical University, Taipei, Taiwan

## Abstract

Hydroxamate derivatives have attracted considerable attention due to their broad pharmacological properties and have been extensively investigated. We recently demonstrated that WMJ-S-001, a novel aliphatic hydroxamate derivative, exhibits anti-inflammatory and anti-angiogenic activities. In this study, we explored the underlying mechanisms by which WMJ-S-001 induces HCT116 colorectal cancer cell death. WMJ-S-001 inhibited cell proliferation and induced cell apoptosis in HCT116 cells. These actions were associated with AMP-activated protein kinase (AMPK) and p38 mitogen-activated protein kinase (MAPK) activation, p53 phosphorylation and acetylation, as well as the modulation of p21^cip/Waf1^, cyclin D1, survivin and Bax. AMPK-p38MAPK signaling blockade reduced WMJ-S-001-induced p53 phosphorylation. Transfection with AMPK dominant negative mutant (DN) reduced WMJ-S-001’s effects on p53 and Sp1 binding to the *survivn* promoter region. Transfection with HDAC3-Flag or HDAC4-Flag also abrogated WMJ-S-001’s enhancing effect on p53 acetylation. WMJ-S-001’s actions on p21^cip/Waf1^, cyclin D1, survivin, Bax were reduced in p53-null HCT116 cells. Furthermore, WMJ-S-001 was shown to suppress the growth of subcutaneous xenografts of HCT116 cells *in vivo*. In summary, the death of HCT116 colorectal cancer cells exposed to WMJ-S-001 may involve AMPK-p38MAPK-p53-survivin cascade. These results support the role of WMJ-S-001 as a potential drug candidate and warrant the clinical development in the treatment of cancer.

Colorectal cancer (CRC) is one of the most prevalent and lethal malignancies, affecting approximately 800,000 individuals each year worldwide^1^. Surgical resection with radio- and/or chemo-therapy is the preferred strategy in the treatment of CRC^2^. Despite advances in anti-cancer drug discovery and development in the last few decades, the prognosis for patients with advanced CRC remains largely unchanged. Therefore, there is an ongoing urgent need for novel drugs in the treatment of CRC.

Recent development in drug discovery has highlighted the diverse biological and pharmacological properties of a key pharmacophore, hydroxamate^3^. Previous studies have demonstrated the potential of hydroxamate derivatives as anti-infectious^4^, anti-inflammatory^5^,^6^, or anti-angiogenic^7^,^8^ agents. It also appears to have anti-tumor properties^7^,^9^,^10^,^11^, although the exact mechanisms remain unclear. Suberoylanilide hydroxamate (vorinostat, SAHA), was recently approved for the treatment of cutaneous T cell lymphoma (CTCL)^12^. Other hydroxamate-based compounds such as panobinostat (LBH589)^13^ and belinostat (PXD101)^14^ also exhibit anti-tumor activities and are currently undergoing clinical trials. These observations suggest that additional hydroxamate derivatives may possess anti-tumor functions capable of therapeutic applications, and are worthy of further development.

We recently synthesized a series of aliphatic hydroxamate derivatives, the WMJ-S compounds, and evaluated their anti-inflammatory^5^ and anti-angiogenesis^7^ properties. This study aims to explore WMJ-S-001’s anti-tumor activity and identify the mechanisms by which it induces HCT116 colorectal cancer cell death.

Apoptosis plays an important role in tissue homeostasis. Any impairment of its regulation may promote tumor formation and malignant progression. As such, alteration in Bcl-2 family proteins, which are the fundamental regulators of intrinsic apoptotic pathway, can contribute to aberrantly increased cancer cell survival^15^. Another important group of apoptosis regulators is the inhibitor of apoptosis protein (IAP) family^16^. Survivin, an IAP family protein, is highly expressed in most cancers and associated with chemotherapy resistance, increased tumor recurrence, and reduced survival rates in patients with CRC^17^,^18^. Survivin also plays an essential role in regulating mitosis and cell division^19^, and has thus emerged as a broader regulator of cellular homeostasis that has been implicated in tumorigenesis^16^,^19^,^20^. Recent studies suggested that survivin may be an independent prognosis factor and a promising target for cancer therapy^19^,^21^,^22^. We recently demonstarted that survivin down-regulation leads to colorectal cancer cell death^23^,^24^. The regulation of survivin expression mainly occurs at the transcriptional level. Many transcription factors including Sp1, c-myc and STAT3 have been shown to contribute to the induction of survivin^25^. Tumor suppressor p53, however, may counteract the binding of Sp1 to the promoter region and, thereby, suppress survivin expression^23^,^26^,^27^.

There is increasing evidence that AMP-activated protein kinase (AMPK) signaling plays regulatory roles in cell survival, growth, and tumorigenesis^28^,^29^. AMPK activation suppresses cell proliferation and induces apoptosis in various types of malignant cells such as glioblastoma cells^30^, prostate cancer cells^28^, gastric cancer cells^29^ and colorectal cancer cells^23^. This inhibition occurs through various mechanisms, including increased expression of the cell cycle regulatory protein, p21^cip/Waf1^^31^, down-regulation of cyclin^29^ and activation of p38MAPK signaling^32^. We aimed to determine whether AMPK signaling cascade contributes to WMJ-S-001’s actions in inducing HCT116 colorectal cancer cell death.

## Results

### WMJ-S-001 inhibited cell proliferation and induced cell apoptosis in HCT116 cells

**Figure 1:**
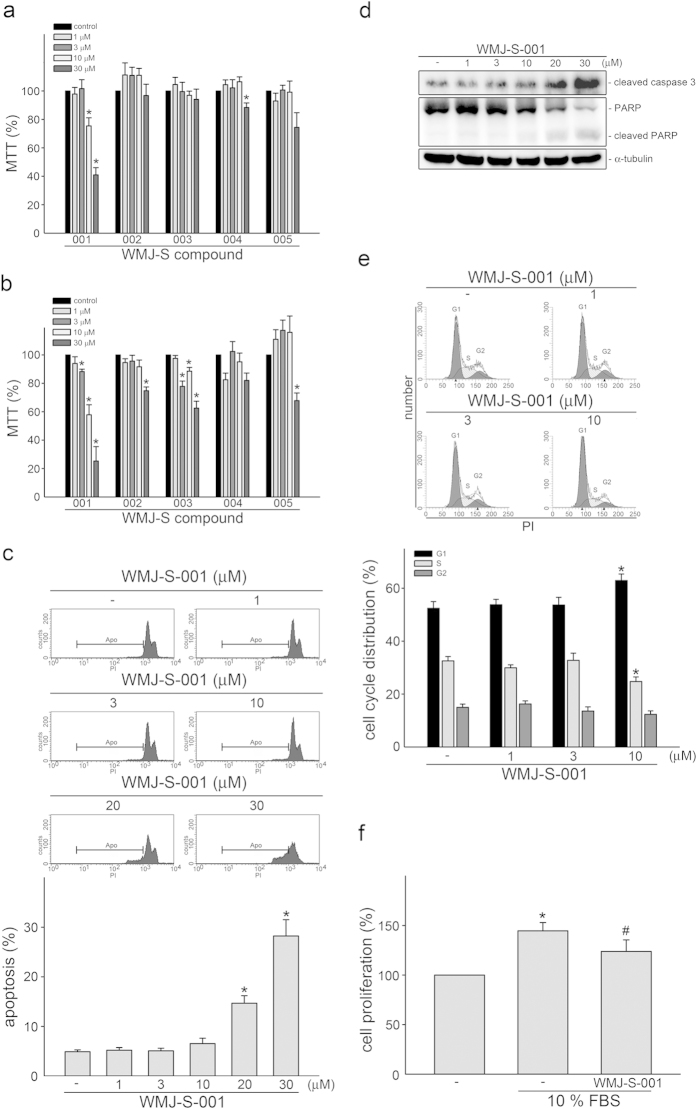
Effects of WMJ-S compounds on cell viability and proliferation in HCT116 cells. Cells were treated with vehicle or WMJ-S-001~005 at indicated concentrations for 24 (**A**) or 48 h (**B**). Cell viability was then determined by an MTT assay. Each column represents the mean ± S.E.M. of at least five independent experiments performed in triplicate (*p < 0.05, compared with the control group). (**C**) Cells were treated with vehicle or WMJ-S-001 at indicated concentrations for 24 h. The percentage of apoptotic cells (Apo) was then analyzed by flow cytometric analysis as described in the *Materials and methods* section. Results shown are representative of three independent experiments. (**D**) Cells were treated as in (**C**), the extent of cleavage caspase 3 and PARP were then determined by immunoblotting. Results shown are representative of five independent experiments. The full-length blot is presented in Supplementary Figure 1. (**E**) Cells were treated as in (**C**), the percentage of cells in G0/G1, S, and G2/M phases was then analyzed by flow-cytometric analysis. Each column represents the mean ± S.E.M. of five independent experiments (*p < 0.05, compared with the control group). (**F**) Cells underwent mitogenic quiescence by serum starvation for 24 h. After starvation, cells were subsequently stimulated with serum (10% FBS) in the presence or absence of WMJ-S-001 for another 48 h. Cell proliferation was then determined as described in the *Materials and methods* section. Each column represents the mean ± S.E.M. of three independent experiments performed in triplicate (*p < 0.05, compared with the control group; ^#^*p *< 0.05, compared with the group treated with 10% FBS alone).

We previously synthesized a series of aliphatic hydroxamate derivatives, the WMJ-S compounds^5^,^7^. To investigate whether these WMJ-S compounds (WMJ-S-001~005) exhibit anti-tumor activites, MTT assay was employed to assess their effects on cell viability in HCT116 colorectal cancer cells. As shown in Fig. 1a, WMJ-S-001 concentration-dependently decreased cell viability of HCT116 cells after 24 h exposure. Longer exposure to WMJ-S-001 (48 h) further decreased HCT116 cell viability (Fig. 1b). In contrast, treatment of cells with WMJ-S-002, WMJ-S-003, WMJ-S-004, or WMJ-S-005 for 24 h only slightly affected cell viability (Fig. 1a). We sought to further investigate the mechanisms of HCT116 cell death after exposure to WMJ-S-001 in the following experiments. To determine whether decreased HCT116 cell viability in the presence of WMJ-S-001 was a result of cell apoptosis, flow cytometric analysis with propidium iodide (PI) labeling was used. As shown in Fig. 1c, WMJ-S-001, at concentrations higher than 10 μM (20 and 30 μM), significantly increased the percentage of PI-stained cells in the apoptotic region (Apo, sub-G1/G1 peak). We next determined whether WMJ-S-001 activates caspase 3. As shown in Fig. 1d, WMJ-S-001 exposure led to an increase in the cleaved (active) form of caspase 3, and resultant cleavage of PARP, a selective caspase 3 substrate.

Flow-cytometric analysis with PI labeling to determine whether WMJ-S-001 at concentrations of 10 μM or lower affects cell cycle progression. As shown in Fig. 1e, the percentage of PI-stained cells in the S region was significantly decreased after 24 h treatment with WMJ-S-001. These effects were accompanied by a concomitant increase in the percentage of PI-stained cells in the G1 region (Fig. 1e). BrdU labeling analysis was employed to confirm whether WMJ-S-001 inhibits HCT116 cell proliferation. Cells were starved with serum free medium for 24 h, and then incubated in serum (10% FBS)-containing medium in the absence or presence of WMJ-S-001 for another 24 h. As shown in Fig. 1f, treatment of cells with WMJ-S-001 (10 μM) significantly decreased serum-induced cell proliferation of HCT116 cells. Taken together, these results suggest that both suppression of cell proliferation and induction of apoptosis contribute to the anti-tumor actions of WMJ-S-001 in HCT116 colorectal cancer cells.

### WMJ-S-001 activated p53 and modulated protein levels of p21, cyclin D1, survivin and Bax in HCT116 cells

**Figure 2:**
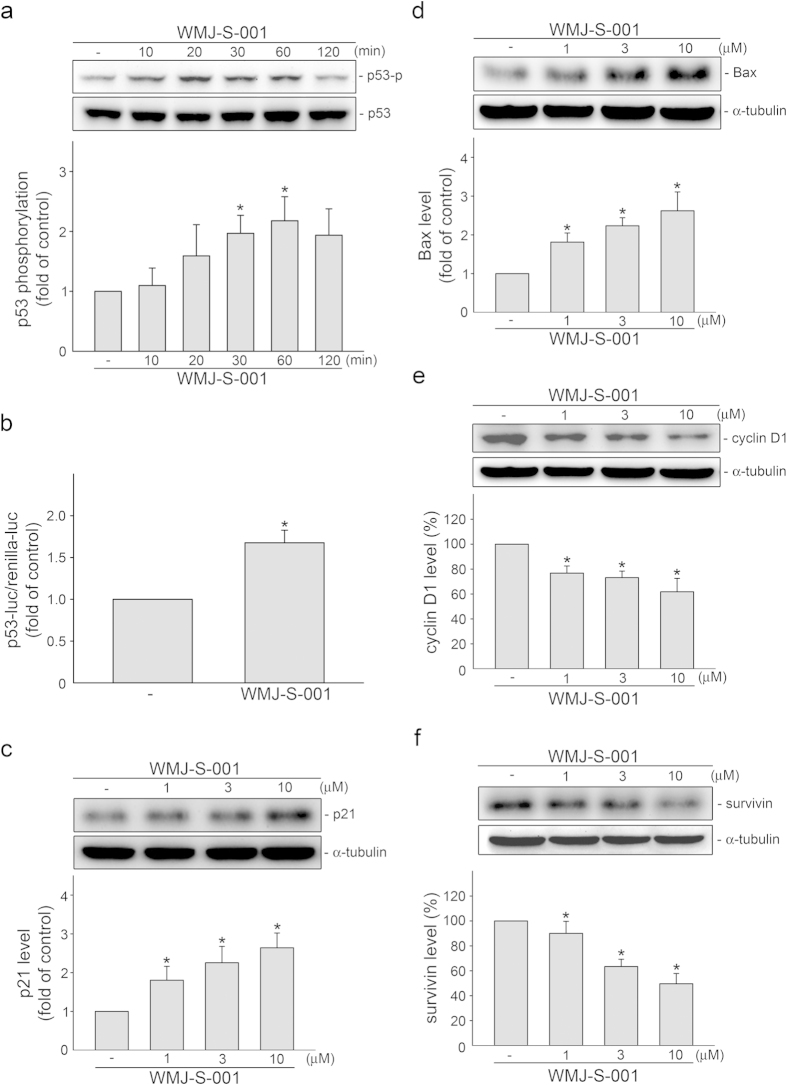
WMJ-S-001 activated p53 and affected the protein levels of p21^cip/Waf1^, Bax, cyclin D1 and survivin in HCT116 cells. (**A**) Cells were treated with vehicle or WMJ-S-001 at 10 μM for various time periods as indicated. The phosphorylation status of p53 were then determined by immunoblotting. Compiled results are shown at the bottom of the chart. Each column represents the mean ± S.E.M. of five independent experiments. The full-length blot is presented in Supplementary Figure 2a (*p < 0.05, compared with the control group). (**B**) Cells were transiently transfected for 48 h with PG13-luc and renilla-luc. After transfection, cells were treated with vehicle or 10 μM WMJ-S-001 for another 24 h. Each column represents the mean ± S.E.M. of four independent experiments (*p < 0.05, compared with the control group). (**C**–**F**) Cells were treated for 24 h with vehicle or WMJ-S-001 at 1, 3 and 10 μM. Protein levels of p21^cip/Waf1^ (**C**), Bax (**D**), cyclin D1 (**E**) and survivin (**F**) were then determined by immunoblotting. Compiled results are shown at the bottom of the chart. Each column represents the mean ± S.E.M. of at least four independent experiments. The full-length blot is presented in Supplementary Figure 2b, 2c, 2d and 2e (*p < 0.05, compared with the control group).

The loss of transcription factor p53 is the most common genetic abnormality found in approximately half of human cancers^33^,^34^,^35^. p53 modulates the expression of target genes, leading to diverse cellular responses including cell cycle arrest and apoptosis^36^,^37^. To explore the role of p53 in WMJ-S-001’s actions in HCT116 cells, we examined the effects of WMJ-S-001 on p53 Ser15 phosphorylation, which is a key step in p53 activation^38^. As shown in Fig. 2a, WMJ-S-001 exposure was associated with an increase in p53 Ser15 phosphorylation in a time-dependent manner. We also examined whether p53 transactivity is increased in cells exposed to WMJ-S-001 using a reporter construct containing a p53 DNA-binding site upstream of a basal promoter linked to a luciferase reporter gene (PG13-luc). As shown in Fig. 2b, cells treated with WMJ-S-001 for 24 h had a significant increase in PG13-luciferase activity. These results suggest that WMJ-S-001 activates p53 in HCT116 cells. P53 modulates several downstream target genes such as p21^cip/Waf1^^39^, Bax^40^, survivin^23^, and cyclin D1, all of which play essential role in cell cycle or apoptosis. We therefore examined whether WMJ-S-001 had any effects on these proteins in HCT116 cells. Immunoblotting analysis demonstrated that p21^cip/Waf1^ (Fig. 2c) and Bax (Fig. 2d) levels were increased, while cycin D1 (Fig. 2e) and survivin (Fig. 2f) were decreased in HCT116 cells exposed to WMJ-S-001.

### p53 in WMJ-S-001’s actions in HCT116 cells

**Figure 3:**
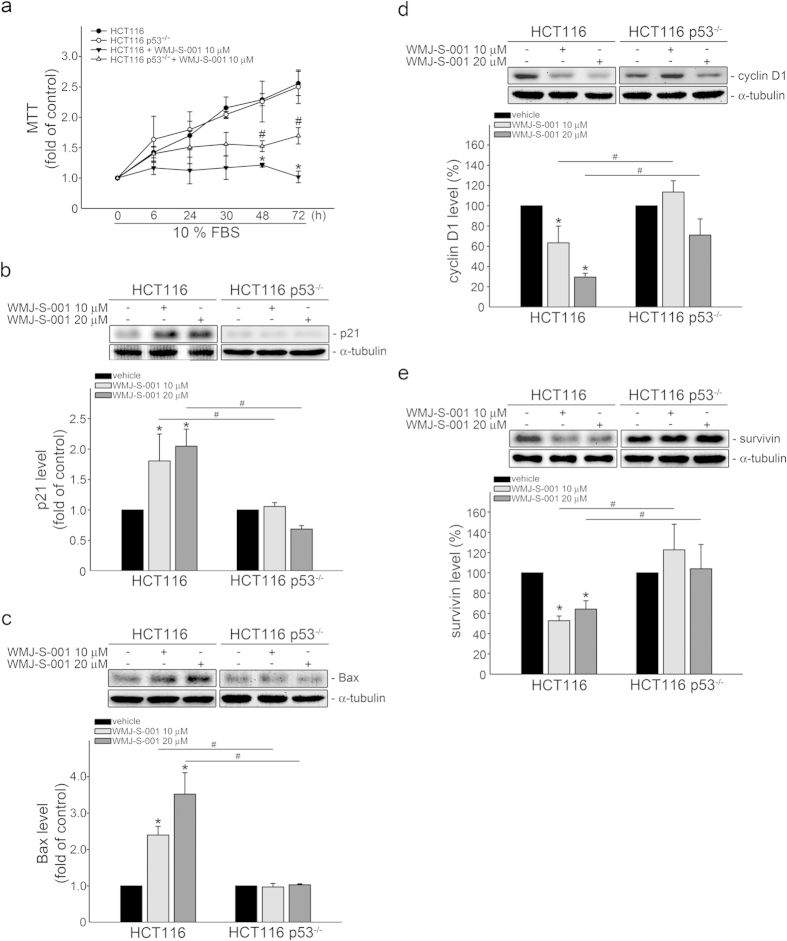
p53 in WMJ-S-001-induced cell apoptosis in HCT116 cells. (**A**) HCT116 and p53 null HCT116 p53^−/−^ cells were starved in serum-free McCoys 5A medium for 24 h. After starvation, cells were treated with vehicle or indicated concentrations of WMJ-S-001 in the presence of 10% FBS for indicated time periods. Cell viability was determined by MTT assay. Each column represents the mean ± SEM of five independent experiments performed in duplicate (*p < 0.05, compared with the HCT116 control group; ^#^*p *< 0.05, compared with the HCT116 group in the presence of WMJ-S-001). (B-E) HCT116 and p53 null HCT116 p53^−/−^ cells were treated with vehicle or WMJ-S-001 at indicated concentrations for 24 h. Protein levels of p21^cip/Waf1^ (**B**), Bax (**C**), cyclin D1 (**D**) and survivin (**E**) were then determined by immunoblotting. Each column represents the mean ± S.E.M. of three independent experiments. The full-length blot is presented in Supplementary Figure 3a, 3b, 3c and 3d (*p < 0.05, compared with the control group; ^#^*p *< 0.05, compared with the WMJ-S-001-treated group).

To further confirm the causal role of p53 in decreasing cell proliferation in HCT116 cells exposed to WMJ-S-001, we compared HCT116 cells that retain wild-type (wt) p53 (HCT116) with their isogenic derivatives, in which the p53 gene had been somatically knocked out (HCT116 p53^−/−^). As shown in Fig. 3a, WMJ-S-001 inhibited serum-induced proliferation in HCT116 cells as well as in HCT116 p53^−/−^ cells. However, the inhibitory effect of WMJ-S-001 on HCT116 p53^−/−^ cells was less pronounced (Fig. 3a). WMJ-S-001’s effects on p21^cip/Waf1^ (Fig. 3b), Bax (Fig. 3c), cycin D1 (Fig. 3d) and survivin (Fig. 3e) levels were also reduced in HCT116 p53^−/−^ cells as compared with HCT116 cells. These results suggest that p53 activation contributes to WMJ-S-001’s effects on HCT116 cell proliferation and survival.

### AMPK-p38MAPK signaling contributes to WMJ-S-001-induced p53 activation

**Figure 4:**
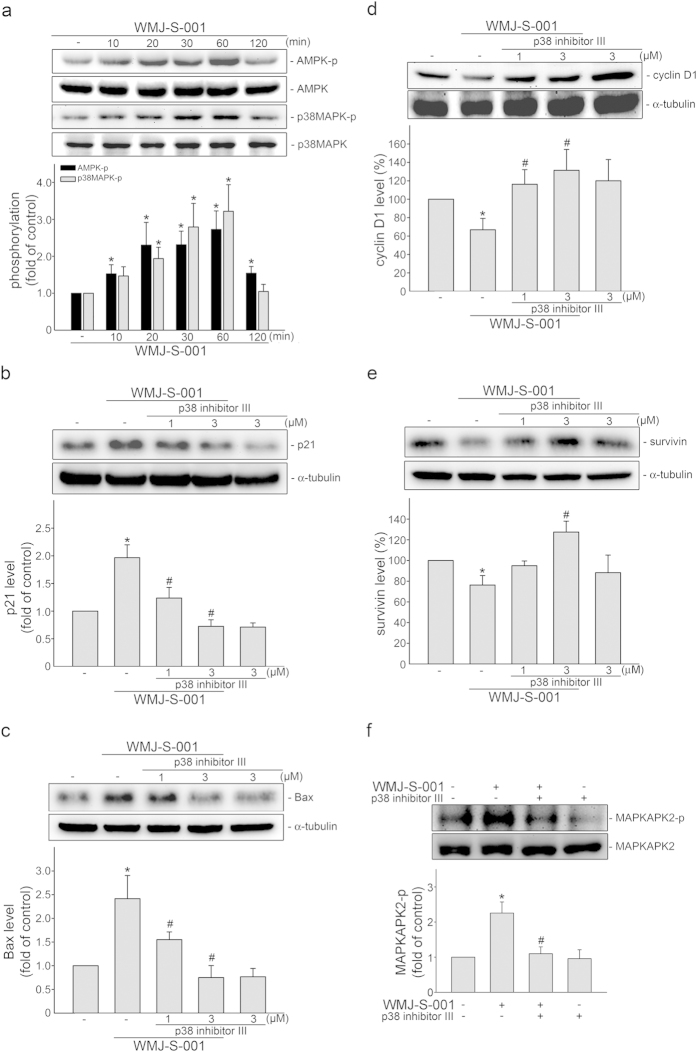
p38MAPK in WMJ-S-001’s actions in HCT116 cells. (**A**) Cells were treated with vehicle or WMJ-S-001 10 μM for indicated time periods. The phosphorylation status of AMPK and p38MAPK were then determined by immunoblotting. The compiled results of AMPK and p38MAPK phosphorylations are shown. Each column represents the mean ± S.E.M. of four independent experiments. The full-length blot is presented in Supplementary Figure 4a (*p < 0.05, compared with the control group). (**B**−**E**) Cells were pretreated for 30 min with vehicle or p38MAPK inhibitor III (1, 3 μM), followed by the treatment with 10 μM WMJ-S-001 for another 24 h. The protein levels of p21^cip/Waf1^ (**B**), Bax (**C**), cyclin D1 (**D**) and survivin (**E**) were then determined by immunoblotting. Complied results are shown at the bottom of the chart. Each column represents the mean ± S.E.M. of three independent experiments. (**F**) Cells were pretreated for 30 min with vehicle or p38MAPK inhibitor III (3 μM), followed by the treatment with 10 μM WMJ-S-001 for another 30 min. The phosphorylation status of MAPKAPK-2 was then determined by immunoblotting. The compiled results of MAPKAPK-2 phosphorylations are shown. Each column represents the mean ± S.E.M. of six independent experiments. The full-length blot is presented in Supplementary Figure 4b–f (*p < 0.05, compared with the control group; ^#^*p *< 0.05; compared with the WMJ-S-001-treated group).

We next explored the signaling cascades that may contribute to WMJ-S-001-induced p53 activation in HCT116 cells. AMPK is one of the upstream protein kinases that phosphorylate p53^41^, which then contributes to AMPK-mediated cell cycle arrest and apoptosis^42^,^43^,^44^. We also previously demonstrated that p38MAPK activates p53, leading to subsequent cell death in cerebral endothelial cells^40^, glioma cells^26^ and colorectal cancer cells^23^. Moreover, AMPK-p38MAPK signaling cascade contributes to hydroxamate derivative trichostatin A (TSA)-induced survivin down-regulation and cell death in colorectal cancer cells^24^. We first examined whether AMPK and p38MAPK phosphorylation are altered in HCT116 cells after WMJ-S-001 exposure. As shown in Fig. 4a, WMJ-S-001 caused an increase in AMPK phosphorylation in a time-dependent manner. p38MAPK phosphorylation is also increased in cells exposed to WMJ-S-001 (Fig. 4a). In contrast, p38MAPK inhibitor III significantly suppressed WMJ-S-001-induced p21^cip/Waf1^ (Fig. 4b) and Bax (Fig. 4c) expression. p38MAPK inhibitor III also restored WMJ-S-001-decreased cycin D1 (Fig. 4d) and survivin (Fig. 4e) levels in HCT116 cells. p38MAPK inhibitor III, similar to SB203580, is a selective ATP-competitive p38 MAPK inhibitor^45^. Kumar S. *et al*.^46^ previously demonstrated that SB203580 has barely effects on p38MAPK phosphorylation induced by upstream kinases. However, SB203580 potently inhibited p38MAPK activity as demonstrated by the inhibition of the activation of MAPKAPK-2, a specific physiological substrate of p38MAPK^46^. Confirming the effects of p38MAPK signaling blockade, a marked reduction in WMJ-S-001-induced MAPKAPK-2 phosphorylation was observed in HCT116 cells treated with 3 μM p38MAPK inhibitor III (Fig. 4f).

**Figure 5:**
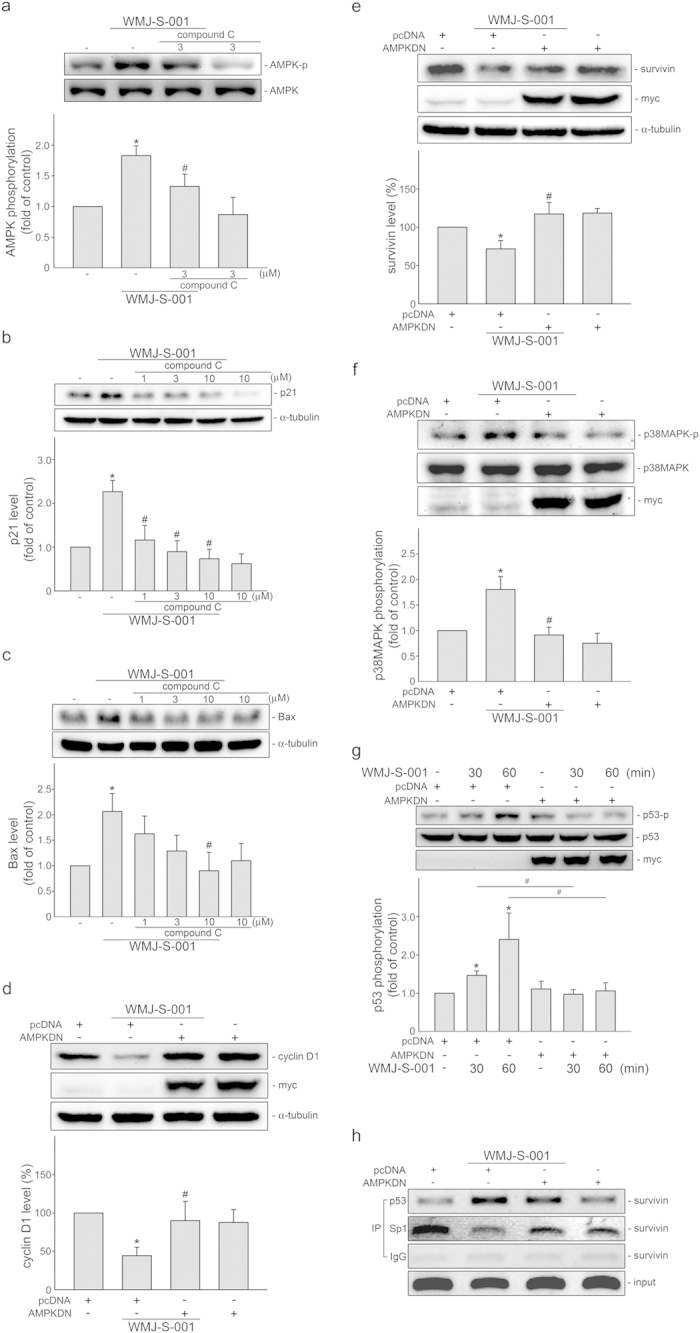
AMPK-p38MAPK signaling contributes to WMJ-S-001-induced p53 activation in HCT116 cells. (**A**−**C**) Cells were pretreated for 30 min with vehicle or compound c followed by the treatment with 10 μM WMJ-S-001 for another 30 min (**A**) or 24 h (**B**,**C**). The phosphorylation status of AMPK (**A**), the protein levels of p21^cip/Waf1^ (**B**) and Bax (**C**) were then determined by immunoblotting. Compiled results are shown at the bottom of the chart. Each column represents the mean ± S.E.M. of at least four independent experiments. The full-length blot is presented in Supplementary Figure 5a–c (*p < 0.05, compared with the control group; ^#^*p *< 0.05; compared with the WMJ-S-001-treated group). (**D**−**G**) Cells were transiently transfected for 48 h with pcDNA or AMPKDN and then treated with WMJ-S-001 (10 μM) for another 24 h (D-E), 30 min (**F**) or 60 min (**G**). Protein levels of cyclin D1 (**D**) and survivin (**E**), the phosphorylation status of p38MAPK (**F**) and p53 (**G**) were then determined by immunoblotting. Compiled results are shown at the bottom of the chart. The extent of myc-tagged AMPKDN was also determined by immunoblotting using anti-myc tag antibody. Each column represents the mean ± S.E.M. of four independent experiments. The full-length blot is presented in Supplementary Figure 5d–g. (*p < 0.05, compared with the pcDNA transfection (mock transfection) group treated with vehicle; ^#^*p *< 0.05, compared with the pcDNA transfection group treated with WMJ-S-001). WMJ-S-001). (**H**) Cells were transiently transfected for 48 h with pcDNA or AMPKDN and then treated with WMJ-S-001 (10 μM) for another 2 h. ChIP assay was then performed as described in the *Materials and methods* section. Typical traces representative of three independent experiments with similar results are shown.

Similarly, an AMPK inhibitor, compound C, not only inhibited WMJ-S-001-induced AMPK phosphorylation (Fig. 5a), but also significantly suppressed WMJ-S-001-induced p21^cip/Waf1^ (Fig. 5b) and Bax (Fig. 5c). WMJ-S-001’s negative impacts on cyclin D1 (Fig. 5d) and survivin (Fig. 5e) were reduced in cells transfected with AMPK dominant negative mutant (DN). Furthermore, AMPK-DN significantly suppressed p38MAPK (Fig. 5f) and p53 (Fig. 5g) phosphorylation in cells exposed to WMJ-S-001.

p53 can lead to suppression of survivin expression by preventing Sp1 binding to the survivin promoter region^25^,^26^. A ChIP experiment was conducted to determine whether p53 or Sp1 is recruited to the endogenous *survivin* promoter region in response to WMJ-S-001. Primers encompassing the survivin promoter region (−264 to −37) containing putative p53 and Sp1 binding sites were used. As shown in Fig. 5h, the binding of p53 to the *survivin* promoter region (−264/−37) increased after 2 h of WMJ-S-001 exposure, and this was accompanied by a decrease in Sp1 binding to the promoter region. WMJ-S-001’s effects on p53 and Sp1 binding to the *survivin* promoter region were reduced in cells transfected with AMPK-DN (Fig. 5h).

### HDAC inhibition contributes to WMJ-S-001’s actions in HCT116 cells

**Figure 6:**
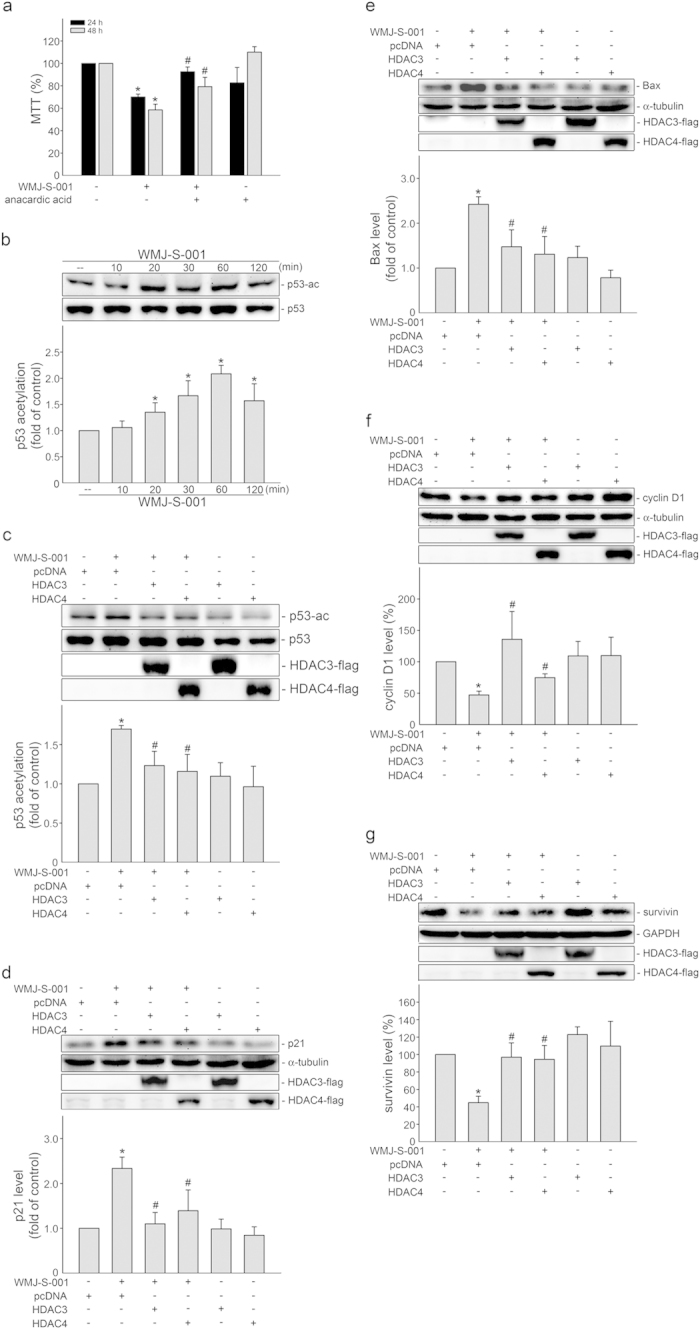
HDACs inhibition in WMJ-S-001’s actions in HCT116 cells. (**A**) Cells were pretreated for 30 min with vehicle or anacardic acid, followed by the treatment with 10 μM WMJ-S-001 for another 24 h or 48 h. Cell viability was then determined by an MTT assay. Each column represents the mean ± S.E.M. of three independent experiments performed in duplicate (*p < 0.05, compared with the control group; ^#^*p *< 0.05, compared with the WMJ-S-001-treated group). (**B**) Cells were treated with vehicle or WMJ-S-001 at 10 μM for various time periods as indicated. The acetylation status of p53 were then determined by immunoblotting. Compiled results are shown at the bottom of the chart. Each column represents the mean ± S.E.M. of five independent experiments. The full-length blot is presented in Supplementary Fig. 6a (*p < 0.05, compared with the control group). (**C**−**G**) Cells were transiently transfected for 48 h with pcDNA, HDAC3-flag, or HDAC4-flag and then treated with WMJ-S-001 (10 μM) for another 60 min (**C**) or 24 h (**D**−**G**). The phosphorylation status of p53 (**C**), the protein levels of p21^cip/Waf1^ (**D**), Bax (**E**), cyclin D1 (**F**) and survivin (**G**) were then determined by immunoblotting. The extent of flag tagged HDAC3 and HDAC4 were determined by immunoblotting using anti-flag tag antibody. Compiled results are shown at the bottom of the chart. Each column represents the mean ± S.E.M. of at least four independent experiments. The full-length blot is presented in Supplementary Fig. 6b–f (*p < 0.05, compared with the pcDNA transfection (mock transfection) group treated with vehicle; ^#^*p* < 0.05, compared with the pcDNA transfection group treated with WMJ-S-001).

The balance between protein acetylation and deacetylation is regulated by histone acetyltransferases (HATs) and histone deacetylases (HDACs)^47^,^48^. Hydroxamate derivatives have been reported to inhibit histone deacetylase (HDAC) activity, resulting in increased acetylation levels of cellular proteins^7^,^10^,^11^. We therefore assessed whether alterations in protein acetylation levels contribute to decreased HCT116 cell viability in the presence of WMJ-S-001. As shown in Fig. 6a, anacardic acid, a histone acetylase (HAT) inhibitor, significantly restored cell viability in WMJ-S-001-stimulated HCT116 cells. We examined whether WMJ-S-001 induces p53 acetylation in HCT116 cells. As shown in Fig. 6b, WMJ-S-001 time-dependently increased p53 acetylation. Transfection of cells with Flag-tagged HDAC3 (HDAC3-Flag, a class I HDAC) or Flag-tagged HDAC4 (HDAC4-Flag, a class II HDAC) suppressed WMJ-S-001-induced p53 acetylation (Fig. 6c). In addition, both HDAC3-Flag and HDAC4-Flag were effective in suppressing WMJ-S-001-elevated p21^cip/Waf1^ (Fig. 6d) and Bax (Fig. 6e) levels. HDAC3-Flag or HDAC4-Flag also restored WMJ-S-001-decreased cyclin D1 (Fig. 6f) and survivin (Fig. 6g) levels. These results support a causal role of HDACs inhibition in WMJ-S-001-induced p53 acetylation and subsequent cellular events in HCT116 cells.

**Figure 7:**
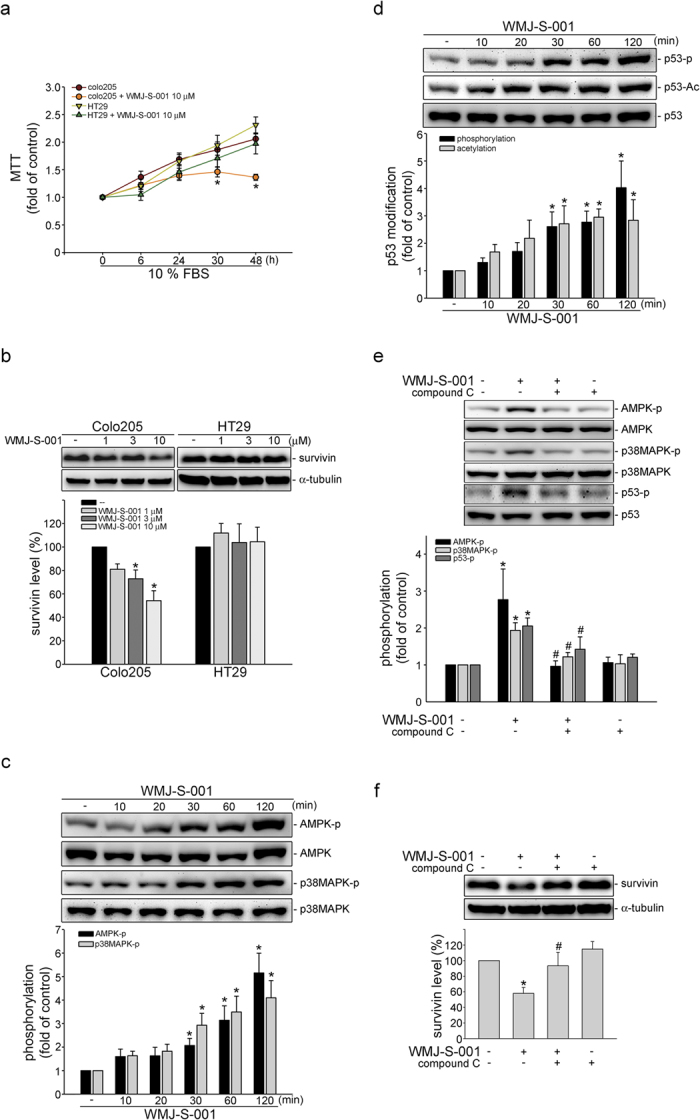
WMJ-S-001 activated AMPK-p38MAPK-p53 signaling and decreased survivin level in Colo205 colorectal cancer cells. (**A**) HT29 and Colo205 cells were starved in serum-free DMEM (HT29) or RPMI1640 (Colo205) medium for 24 h. After starvation, cells were treated with indicated concentrations of WMJ-S-001 in the presence of 10% FBS for indicated time periods. Cell viability was determined by MTT assay. Each column represents the mean ± SEM of five independent experiments performed in duplicate (*p < 0.05, compared with the control group). (**B**) Cells were treated for 24 h with WMJ-S-001 (1–10 μM). Protein levels of survivin was then determined by immunoblotting. Each column represents the mean ± S.E.M. of four independent experiments. The full-length blot is presented in Supplementary Fig. 7a (*p < 0.05, compared with the control group) (**C**) Colo205 cells were treated with WMJ-S-001 (10 μM) for indicated time periods. The phosphorylation status of AMPK and p38MAPK were then determined by immunoblotting. Each column represents the mean ± S.E.M. of eight independent experiments. The full-length blot is presented in Supplementary Fig. 7b (*p < 0.05, compared with the control group). (**D**) Colo205 cells were treated with WMJ-S-001 at 10 μM for indicated time periods. The phosphorylation and acetylation status of p53 were then determined by immunoblotting. Each column represents the mean ± S.E.M. of six independent experiments. The full-length blot is presented in Supplementary Fig. 7c (*p < 0.05, compared with the control group). (**E**−**F**) Colo205 cells were pretreated for 30 min with vehicle or compound c (3 μM), followed by the treatment with 10 μM WMJ-S-001 for another 30 min (**E**) or 24 h (**F**). The phosphorylation status of AMPK, p38AMPK and p53 (**E**) or the survivin levels (**F**) were then determined by immunoblotting. Each column represents the mean ± S.E.M. of at least four independent experiments. The full-length blot is presented in Supplementary Fig. 7d,e (*p < 0.05, compared with the control group; ^#^*p *< 0.05, compared with the WMJ-S-001-treated group).

In addition to HCT116 cells, we also determined the WMJ-S-001’s effects on growth of another two colorectal cancer cell lines, HT29 and Colo205 cells. HT29 is a p53 mutant cell line^49^ while Colo205 cell retains functional p53^50^. As shown in Fig. 7a, WMJ-S-001 significantly inhibited serum-induced proliferation in Colo205 cells. However, the inhibitory effect of WMJ-S-001 on HT29 cells was less pronounced (Fig. 7a). WMJ-S-001’s effects on survivin (Fig. 7b) levels were also reduced in HT29 cells as compared with Colo205 cells. Moreover, WMJ-S-001 caused increases in AMPK and p38MAPK phosphorylations (Fig. 7c) as well as p53 phosphorylation and acetylation (Fig. 7d) in Colo205 cells. Furthermore, compound C significantly suppressed the phosphorylation of AMPK, p38MAPK and p53 (Fig. 7e) and restored survivin level (Fig. 7f) in Colo205 cells exposed to WMJ-S-001. These results further confirm that AMPK-p38MAPK-p53-survivin cascade contributes to WMJ-S-001’s effects on colorectal cancer cell death.

### WMJ-S-001 attenuated colorectal tumor growth in a murine xenograft model

**Figure 8:**
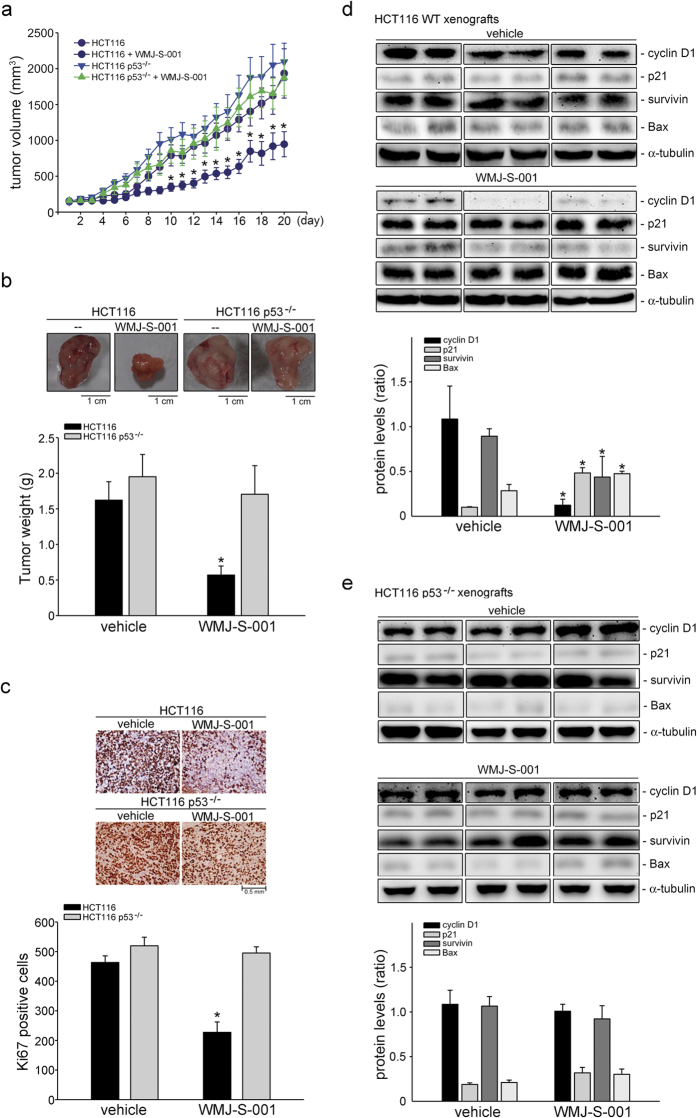
WMJ-S-001 suppressed *in vivo* tumor growth in nude mice. (**A**) Nude mice bearing xenografts of HCT116 or HCT116 p53^−/−^ colorectal cancer cells were treated intraperitoneally with WMJ-S-001 20 mg/kg/day for 20 days. The control group received vehicle only. Tumor volumes were calculated as described in the *Materials and Methods* section. Values represents the mean ± S.E.M. (*p < 0.05 as compared with the vehicle-treated control group, n = 5). (**B**) After 20 days of treatment, mice were sacrificed and tumors were dissected and weighted. Each column represents the mean ± S.E.M. (*p < 0.05 as compared with the vehicle-treated control group, n = 5). (**C**) The proliferative cells in solid tumour sections were stained with anti-Ki67 antibody. Images of immunohistochemical staining representative of at least four independent experiments with similar results are shown. Compiled results are shown at the bottom of the chart. Each column represents the mean ± SEM of five independent experiments. (*p < 0.05, significantly different from the vehicle group. Protein lysates obtained from six randomly selected HCT116 (**D**) or HCT116 p53^−/−^ (**E**) xenograft tumors (three tumors from each group) were subjected to immunoblotting for assessing p21^cip/Waf1^, cyclin D1, survivin, Bax and α-tubulin levels. Compiled results are shown. Values represents the mean ± S.E.M of three tumors from each group performed in duplicate. The full-length blot is presented in Supplementary Fig. 8 (*p < 0.05 as compared with the vehicle-treated control group).

**Figure 9:**
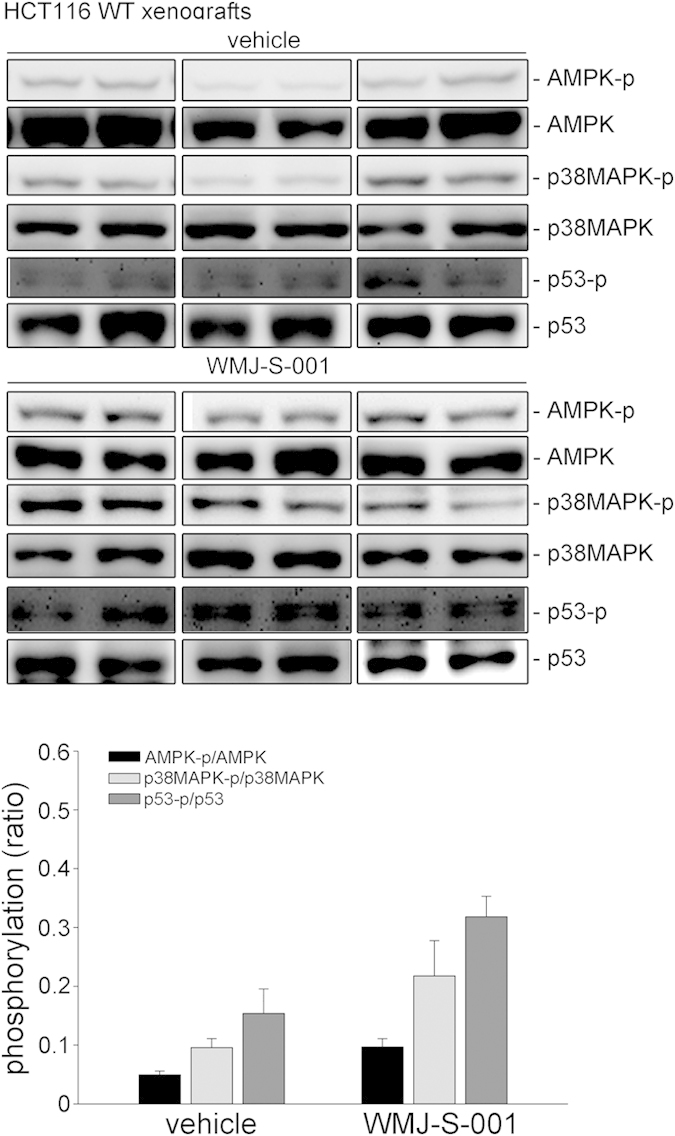
WMJ-S-001 induced AMPK, p38MAPK and p53 phosphorylation in HCT116 xenograft tumors. Protein lysates obtained from six randomly selected HCT116 xenograft tumors (three tumors from each group) were subjected to immunoblotting for assessing the phosphorylation status of AMPK, p38MAPK and p53. Compiled results are shown. Values represents the mean ± S.E.M of three tumors from each group performed in duplicate. The full-length blot is presented in Supplementary Fig. 9 (*p < 0.05 as compared with the vehicle-treated control group).

We used a murine xenograft colorectal tumor model to further investigate the *in vivo* effects of WMJ-S-001. HCT116 or HCT116 p53^−/−^ cells were injected into the flanks of nude mice. After allowing the tumors to grow subcutaneously to an average size of about 150 mm^3^, either vehicle or WMJ-S-001 (20 mg/kg/day) was administered intraperitoneally for 20 days. Mice were sacrificed at the end of the 20-day treatment and tissue samples were collected. WMJ-S-001 markedly reduced HCT116 xenograft tumors growth (Fig. 8a) and tumor weight (Fig. 8b) comparing to the vehicle-treated control group. However, HCT116 p53^−/−^ xenograft tumors growth (Fig. 8a) and tumor weight (Fig. 8b) were barely affected by the presence of WMJ-S-001. We also examined the expression of Ki-67, a cellular marker for proliferation, by immunohistochemistry (IHC) staining to determine whether cell proliferation was suppressed by WMJ-S-001 in dissected tumors. There was a significant decrease in the number of Ki-67-positive cells in WMJ-S-001-treated HCT116 xenograft tumors compared with vehicle-treated tumors, indicative of reduced proliferation (Fig. 8c). However, no significant differences in the number of Ki-67-positive cells were found among the vehicle- and WMJ-S-001-treated HCT116 p53^−/−^ xenografts tumors (Fig. 8c). The protein levels of p21^cip/Waf1^, Bax, cyclinD1, and survivin in the excised tumors were also examined. As shown in Fig. 8d, p21^cip/Waf1^ and Bax levels were elevated while cyclinD1 and survivin levels were decreased in HCT116 xenograft tumors from WMJ-S-001-treated mice. However, WMJ-S-001’s effects on p21^cip/Waf1^, Bax, cycin D1 and survivin levels were reduced in HCT116 p53^−/−^ xenograft tumors as compared with HCT116 xenograft tumors (Fig. 8e). We further examined the phosphorylation status of AMPK, p38MAPK and p53 in the excised HCT116 xenograft tumors. As shown in Fig. 9, The phosphorylation of AMPK, p38MAPK and p53 were elevated in HCT116 xenograft tumors from WMJ-S-001-treated mice. These results suggest that WMJ-S-001 treatment is capable of suppressing tumor growth *in vivo* through, at least in part, regulation of p21^cip/Waf1^, Bax, cyclinD1 and survivin. It also indicated that AMPK-p38MAPK-p53 signaling contributes to WMJ-S-001’s effects on HCT116 xenograft tumors growth.

## Discussion

Colorectal cancer incidence rate increases substantially during these years and CRC remains one of the leading causes of cancer-related deaths^1^. Surgical resection with adjuvant radio- or chemo-therapy is common approach in the treatment of CRC. However, advanced CRC is refractory to most conventional anti-cancer therapies, highlighting the need for novel therapeutic agents or strategies^51^. There is growing evidence suggesting beneficial effects of hydroxamate derivatives in the treatment of cancer^7^,^9^,^10^,^11^, although their underlying anti-tumor mechanisms are incompletely understood. We recently identified a novel aliphatic hydroxamate derivative, WMJ-S-001, which suppresses angiogenesis and tumor growth *in vivo*^7^. In this study, we further demonstrated that WMJ-S-001 activates AMPK-p38MAPK-p53-survivin signaling cascade to induce HCT116 colorectal cancer cell death. Alteration in the acetylation status of cellular proteins including p53 may also contribute to WMJ-S-001’s actions in HCT116 cells.

Hydroxamate derivatives have been reported to increase cell cycle regulator p21^cip/Waf^ and suppress cell proliferation^9^ In keeping with previous observations, we demonstrated that WMJ-S-001 elevates p21^cip/Waf^ level while reducing cyclin D1 levels in HCT116 cells. While WMJ-S-001 at lower concentration (i.e. ≤10 μM) merely suppressed cell cycle progression, at higher concentrations it is capable of inducing caspase 3 activation and subsequent cell apoptosis in HCT116 cells as well as in HUVECs^7^. We noted that WMJ-S-001-modulated p21, cyclin D1, Bax and survivin levels were restored in p53-null HCT116 cells (HCT116 p53^−/−^) and in HCT116 p53^−/−^ xenografts. No significant differences in the number of Ki-67-positive cells and tumor growth were found among the vehicle- and WMJ-S-001-treated HCT116 p53^−/−^ xenografts. These observations indicated that WMJ-S-001’s anti-proliferative and apoptotic actions are attributable to p53 signaling in HCT116 cells. p53 stability and activity are dependent on post-transcriptional modifications such as phosphorylation, sumoylation, ubiquitination and acetylation^38^. Our results indicate that WMJ-S-001 activates AMPK-p38MAPK cascade, leading to p53 phosphorylation. WMJ-S-001 also markedly increased p53 acetylation. It has been reported that p53 phosphorylation promotes the recruitment of cofactors such as CBP, which acetylate p53 and further augment its anti-proliferative ability^52^. It is likely that WMJ-S-001 phosphorylates and thereby acetylates p53 in HCT116 cells. In addtion, HDACs inhibition contributes to WMJ-S-001-induced p53 acetylation.

Excess iron has been reported to be associated with tumorigenesis and metastasis in a variety of human malignancies^53^,^54^. Iron chelation has thus emerged as a novel strategy in improving cancer treatment^55^, and has been shown to increase p53 levels in hepatocellular carcinoma^56^. Excess iron negatively regulates p53 signaling by destabilizing p53 protein via the ubiquitin-proteasome system (UPS) and interfering with its binding to the target DNA^57^. Since hydroxamate derivatives are also known as iron siderophores, it raises the possibility that WMJ-S-001 chelates iron which contributes to the activation of p53. Further investigations are needed to clarify this.

Class I HDAC levels (HDAC1, 2, 3 and 8) are observed in various types of cancers and are associated with a poor prognosis^58^,^59^. We demonstrated that HAT inhibitor anacardic acid restored WMJ-S-001-decreased cell viability in this study. Transfection of HCT116 cells with HDAC3-flag (a class I HDAC) or HDAC4-flag (a class II HDAC) significantly reduced WMJ-S-001’s effects in the acetylation of p53 and the modulation of p21^cip/Waf1^, cyclin D1, *survivin* and Bax levels. These results suggest that alteration of cellular acetylation status plays a causal role in WMJ-S-001-induced colorectal cancer cell death Further investigations are needed to determine whether other HDACs besides HDAC3/4 contribute to the anti-tumor actions of WMJ-S-001.

Similar to previous report^24^, we noted that AMPK-p38MAPK cascade contributes to WMJ-S-001-induced cell death in HCT116 cells. However, the mechanisms of this activation, and whether this leads to autophagy in HCT116 cells, as suggested in another study^60^, remain to be established. In addition to AMPK, recent report has indicated that SHP-1-PP2A-p38MAPK cascade leads to p53 activation and cell death in vascular smooth muscle cells^61^. We previously established that WMJ-S-001 suppression of angiogenesis involves activation of SHP-1 in HUVECs^7^. We also found that SHP-1 inhibitor NSC-87877 reduces WMJ-S- 001’s effects on p53 downstream targets including p21^cip/Waf1^, survivin and Bax in HCT116 cells (unpublished data). These findings suggest that SHP-1 may be causally related to WMJ-S-001-induced p53 activation and subsequent signaling events in HCT116 cells. The link between SHP-1- and AMPK-mediated p53 activation and the differential mechanisms of WMJ-S-001′ actions in driving these two signaling pathways remain to be elucidated. It is likely that these two pathways converge in p53 activation and cell death. Additional works are needed to characterize the relationship between SHP-1 and AMPK signaling in WMJ-S-001-induced HCT116 cell death.

In conclusion, we have shown in the present study that WMJ-S-001 exhibits anti-tumor effects, at least in part, via AMPK-p38MAPK-p53-survivin signaling cascade in HCT116 colorectal cancer cells. Moreover, WMJ-S-001 has additional properties with anti-tumor effects, such as anti-angiogenic^7^ and anti-inflammatory activities^5^. The exact mechanisms of these activities remain to be fully investigated, but together these observations support the potential of WMJ-S-001 as a valuable lead compound in the development of future oncologic therapy.

## Materials and Methods

### Reagents

McCoys 5A, DMEM and RPMI1640 medium and 3-[4, 5-dimethylthiazol-2-yl]-2, 5-diphenyltetrazolium bromide (MTT) were from Sigma-Aldrich (St Louis, MO, USA). TrypLE™ and all cell culture reagents were purchased from Invitrogen (Carlsbad, CA, USA). Fetal bovine serum (FBS) was purchased from Biological Industries (Kibbutz Beit Haemek, Israel). Compound C (6-[4-(2-Piperidin-1-yl-ethoxy)-phenyl)] -3-pyridin-4-yl-pyrrazolo [1,5- -a]-pyrimidine) and a p38MAPK inhibitor III (ML3403, (RS)-{4-[5-(4-Fluorophenyl)- 2-methylsulfanyl-3H- imidazol-4-yl]pyridin-2-yl}-(1-phenylethyl)amine]) were bought from Calbiochem (San Diego, CA, USA). Cell Proliferation ELISA, BrdU assay kit was acquired from Roche (Indianapolis, IN, USA). Antibody against p53, Bax, p21, Sp1 and normal IgG were purchased from Santa Cruz Biotechnology (Santa Cruz, CA, USA). Antibodies against survivin, AMPKα phosphorylated at Thr172 (T172), p53 phosphorylated at Ser15 (S15), p53 acetylated at Lys379 (K379), p38MAPK, p38MAPK phosphorylated at Thr180/Tyr182 (T180/Y182), MAPKAPK-2, MAPKAPK-2 phosphorylated at Thr334 (T334), caspase 3 active form and PARP were purchased from Cell Signaling (Danvers, MA, USA). Anti-mouse and anti-rabbit IgG conjugated horseradish peroxidase antibodies, as well as antibodies against α-tubulin, AMPKα, cyclin D1, DDDDK (Flag) and myc tag were obtained from GeneTex Inc (Irvine, CA, USA). Turbofect^TM^ *in vitro* transfection reagent was purchased from Upstate Biotechnology (Lake Placid, NY, USA). Construct of PG13-luc with p53 binding sites (p53-luc, Addgene plasmid 16642) as described previously^36^ was kindly provided by Dr. Bert Vogelstein. AMPK dominant negative mutant (AMPKDN) was provided by Dr. Morris Birnbaum (HHMI, PA, USA). HDAC3-Flag (Addgene plasmid 13819) and HDAC4-Flag (Addgene plasmid 13821) constructs as described previously^62^ were provided by Dr. Eric Verdin (Department of Medicine, University of California, San Francisco, USA). Renilla-luc, and the Dual-Glo luciferase assay system were purchased from Promega (Madison, WI, USA). All materials for immunoblotting were purchased from GE Healthcare (Little Chalfont, UK). The enhanced chemiluminescence detection kit was from Millipore (Billerica, MA, USA). All other chemicals were obtained from Sigma-Aldrich (St Louis, MO, USA).

### Synthesis of WMJ-S-001

WMJ-S-001 was synthesized as described previously^7^.

### Cell culture

HCT116 cell lines with and without target deletions of p53 (HCT116, p53^+/+^ and HCT116, p53^−/−^) were kindly provided by Dr. Bert Vogelstein^63^. HT29 and Colo205 cell lines were obtained from the American Type Culture Collection (Livingstone, MT, USA). The cells were maintained in McCoys 5A (HCT116), DMEM (HT29) or RPMI1640 (Colo205) containing 10% FBS, 100 U/ml of penicillin G, and 100 μg/ml streptomycin in a humidified 37 °C incubator.

### Cell viability assay

Cell viability was measured by the colorimetric 3-(4,5-dimethylthiazol-2-yl)-2,5-diphenyl tetrazolium bromide (MTT) assay as described previously^7^.

### Cell proliferation assay (BrdU incorporation assay)

HCT116 cells (2 × 10^4^ per well) were seeded in 24-well tissue culture plates and incubated for 24 h. Cells were then starved in serum-free McCoys 5A medium for another 24 h. After starvation, cells were stimulaed with serum (10% FBS) in the absence or presence of WMJ-S-001 for another 24 h. Cell proliferation was then determined using a Cell Proliferation ELISA, BrdU (colorimetric) kit (Roche) based on the colorimetric detection of the incorporation of BrdU, following the manufacturer’s instructions.

### Flow cytometric analysis

HCT116 cells were incubated with WMJ-S-001 at indicated concentrations for 24 h. At the end of the experiments, cells were washed twice with PBS and re-suspended in ice-cold 70% ethanol at 0 °C overnight. Cells were washed with phosphate-citric acid buffer and subsequently stained with propidium iodide (PI) staining buffer containing 0.1% Triton X-100, 100 μg/ml RNase A, and 30 μg/ml PI for 30 min in the dark. Cells were then filtered on a nylon mesh filter. The samples were analyzed using the FACScan and Cellquest program (BD Biosciences, San Jose, CA. USA). The ModFit programs (BD Biosciences, San Jose, CA) were used to determine the percentage of PI-stained cells in the subG1 (Apoptosis, Apo), G0/G1, S or G2/M region.

### Immunoblot analysis

Immunoblot analyses were performed as described previously^7^. Briefly, cells were lysed in an extraction buffer containing 10 mM Tris (pH 7.0), 0.5% NP-40, 140 mM NaCl, 2 mM PMSF, 5 mM DTT, 0.05 mM pepstatin A, and 0.2 mM leupeptin. Samples of equal amounts of protein were subjected to SDS-PAGE and transferred onto a NC membrane (Pall Corporation, Washington, NY, U.S.A.) which was then incubated in a TBST buffer containing 5% non-fat milk. Proteins were visualized by incubating with specific primary antibodies followed by horseradish peroxidase-conjugated secondary antibodies. Immunoreactivity was detected based on enhanced chemiluminescence per the instructions of the manufacturer. Quantitative data were obtained using a computing densitometer with a scientific imaging system (Kodak, Rochester, NY, U.S.A.).

### Transfection in HT116 cells

HCT116 cells (7 × 10^4^ cells per well) were transfected with PG13-luc (p53-luc) plus Renilla-luc for reporter assay or transfected with pcDNA, AMPK dominant negative (AMPKDN), HDAC3-Flag or HDAC4-Flag for immunoblotting analysis using Turbofect reagent (Upstate Biotechnology, Lake Placid, NY) according to manufacturer’s instructions.

### Dual luciferase reporter assay

Cells were transfected with PG13-luc (p53-luc) plus Renilla-luc using Turbofect reagent (Upstate Biotechnology, Lake Placid, NY). Cells with and without treatments were then harvested, and the luciferase activity was determined using a Dual-Glo luciferase assay system kit (Promega) according to manufacturer’s instructions, and was normalized on the basis of Renilla luciferase activity.

### Chromatin immunoprecipitation (ChIP) assay

A ChIP assay was performed as described previously^5^. Briefly, cells were cross-linked with 1% formaldehyde at 37 °C for 10 min and then rinsed with ice-cold PBS. Cells were then harvested in SDS lysis buffer, sonicated ten times for 15 s each, and then centrifuged for 10 min. Supernatants were collected and diluted in ChIP dilution buffer, followed by immunoclearing with gentle rotation with 60 μl protein A-agarose slurry for 1 h at 4 °C. An aliquot of each sample was used as “input” in the PCR analysis. The remainder of the soluble chromatin was incubated at 4 °C overnight with p53, Sp1 antibodies or control normal IgG (Santa Cruz Biotechnology, Santa Cruz, CA, USA). Immune complexes were collected by incubation with 20 μl protein A-Magnetic Beads (Millipore, Billerica, MA, USA) for 2 h at 4 °C with a gentle rotation. The complexes were washed sequentially for 5 min in the following three washing buffers: low salt immune complex washing buffer, high-salt immune complex washing buffer, and LiCl immune complex washing buffer. Precipitates were washed two times with Tris-EDTA buffer. The complexes were then eluted twice with two 100 μl aliquots of elution buffer. The cross-linked chromatin complex was reversed in the presence of 0.2 M NaCl and heating at 65 °C for 4 h. DNA was purified using GP^TM^ DNA purification spin columns (Viogene, New Taipei City, Taiwan). PCR was performed using PCR Master Mix (Promega, Madison, WI, USA), according to the manufacturer’s protocol. Ten percent of the total purified DNA was used for the PCR in a 50-μl reaction mixture. The 228-bp *survivin* promoter fragment between −264 and −37 was amplified using the primer pair, sense: 5′-ttc ttt gaa agc agt cga gg-3′ and antisense: 5′-tca aat ctg gcg gtt aat gg-3′, in 30 cycles of PCR. This was done: at 95 °C for 30 s, at 56 °C for 30 s, and at 72 °C for 45 s. The PCR products were analyzed by 1.5% agarose gel electrophoresis

### Ethic statement

This study was carried out in strict accordance with the recommendations in the Guide for the Care and Use of Laboratory Animals of the National Institutes of Health. The protocols described below were approved by the Taipei Medical University Laboratory Animal Care and Use Committee (Permit Number: LAC-2013-0166). All surgery was performed under sodium pentobarbital anesthesia, and all efforts were made to minimize suffering.

### Mouse xenograft colorectal tumor model

3–5 week old nude_nu/nu_ mice were obtained from BioLasco (Taipei, Taiwan) and housed in clean specific pathogen free (SPF) rooms. HCT116 or HCT116 p53^−/−^ cells were harvested and resuspended in PBS, and 5 × 10^6^ cells in a volume of 250 μl were injected subcutaneously into the right flank of each mouse. Once the tumor reached approximately 150 mm^3^, animals were randomized into the control group and the treatment groups, which received WMJ-S-001 20 mg/kg/day. The treatment was administered intraperitoneally once daily for 20 days. Tumors were measured every day by a digital caliper. Tumor volume was calculated using the formula *V* (mm3) = [*ab*2] × 0.52, where *a* is the length and *b* is the width of the tumor^7^. At the end of treatment, animals were sacrificed and tumors were removed. All protocols were approved by the Taipei Medical University Animal Care and Use Committee.

### Immunohistochemical anylysis

Multiple cryosections were obtained from HCT116 or HCT116 p53^−/−^ tumor xenografts. The proliferative cells (Ki67^+^ area) were determined using a rabbit anti-Ki67 antibody (Novus Biologicals, Littleton, CO, USA) and peroxidase-conjugated goat anti-rabbit antibody (The Jackson Laboratory, Sacramento, CA, USA). Antibody binding was visualized using stable diaminobenzidine. Images were obtained in four different quadrants of each tumor section at ×40 magnification. Measurement of the area of Ki67-stained proliferative cells was done as described previously^7^.

### Statistical analysis

Results are presented as the mean ± S.E. from at least three independent experiments. One-way analysis of variance (ANOVA) was followed by the Newman-Keuls test, when appropriate, to determine the statistical significance of the difference between means. A *p* value of <0.05 was considered statistically significant.

## Additional Information

**How to cite this article**: Huang, Y.-H. *et al*. The effects of a novel aliphatic-chain hydroxamate derivative WMJ-S-001 in HCT116 colorectal cancer cell death. *Sci. Rep*. **5**, 15900; doi: 10.1038/srep15900 (2015).

## Supplementary Material

Supplementary Information
