# Supplementary material for: The effects of a novel aliphatic-chain hydroxamate derivative WMJ-S-001 in HCT116 colorectal cancer cell death
Source: Sci Rep. 2015 Oct 29;5:15900. doi: 10.1038/srep15900 (PMC4625135; doi:10.1038/srep15900)
Supplement: Supplementary Information [file srep15900-s1.pdf]

The effects of a novel aliphatic-chain hydroxamate derivative WMJ-S-001 in HCT116 colorectal cancer cell death

Yu-Han Huang[1], Shiu-Wen Huang[2], Ya-Fen Hsu[3], George Ou[4], Wei-Jan Huang[5*], Ming-Jen Hsu[1,6*]

[1]Graduate Institute of Medical Sciences, College of Medicine, Taipei Medical University, Taipei, Taiwan; [2]Graduate Institute of Pharmacology, College of Medicine, National Taiwan University, Taipei, Taiwan; [3]Division of General Surgery, Department of Surgery, Landseed Hospital, Taoyuan, Taiwan; [4]Department of Medicine, University of British Columbia, Vancouver, British Columbia, Canada; [5]Graduate Institute of Pharmacognosy, Taipei Medical University, Taipei, Taiwan; [6]Department of Pharmacology, School of Medicine, College of Medicine, Taipei Medical University, Taipei, Taiwan

[*]Correspondence should be addressed to:

Dr. Wei-Jan Huang, Graduate Institute of Pharmacognosy, Taipei Medical University, No.250, Wu-hsing St., Taipei 11031, Taiwan; Tel: +886-2-27361661 ext. 6152; Fax: +886-2-27355276; E-mail: wjhuang@tmu.edu.tw

Dr. Ming-Jen Hsu, Department of Pharmacology, School of Medicine, Taipei Medical University, No.250, Wu-hsing St., Taipei 11031, Taiwan; Tel: +886-2-27361661 ext. 3198; E-mail: aspirin@tmu.edu.tw

(Ming-Jen Hsu will communicate with the editorial office and, if necessary, the production office.)

a

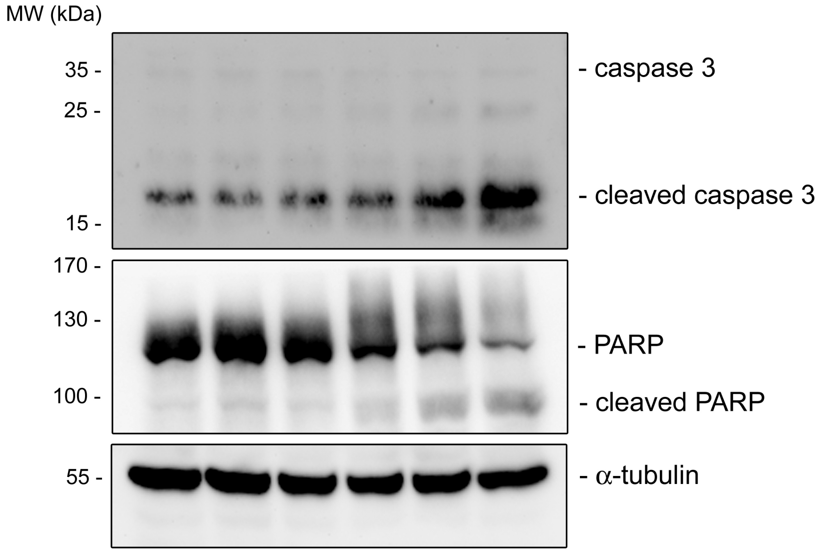
MW (kDa)
35 -
25 -
15 -
170 -
130 -
100 -
55 -
- caspase 3
- cleaved caspase 3
- PARP
- cleaved PARP
- α-tubulin

Supplementary Figure 2

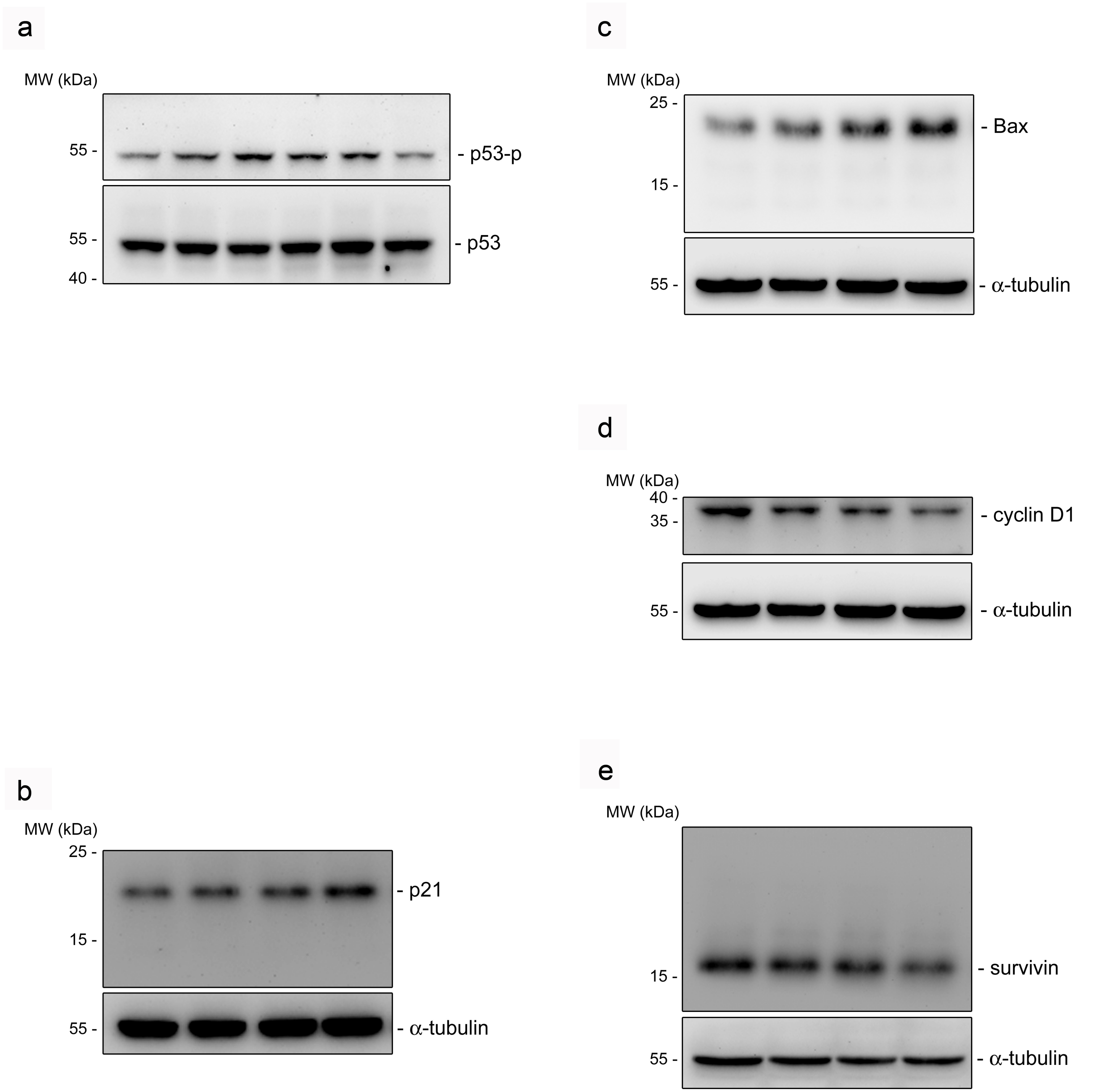

Supplementary Figure 3

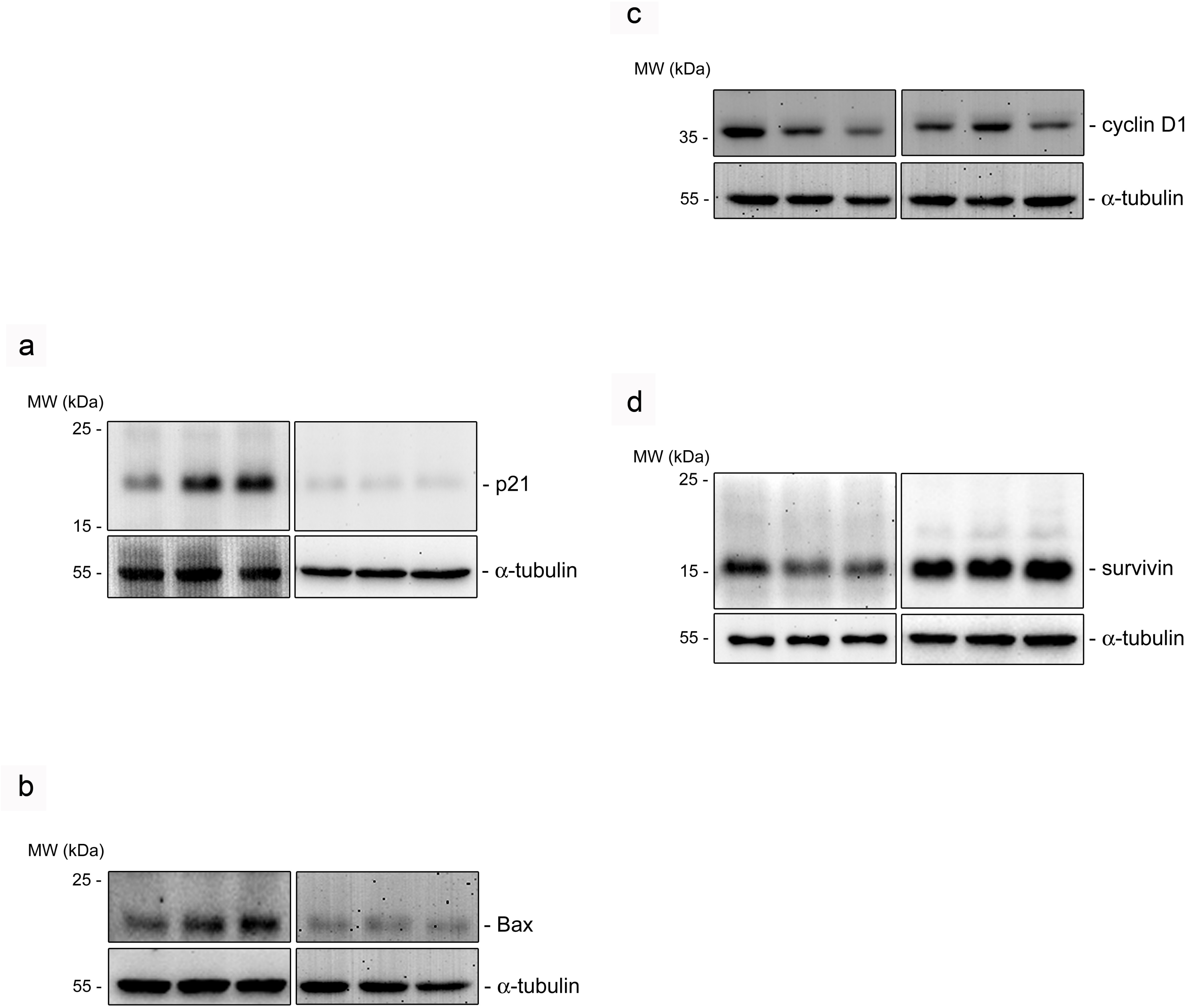

Supplementary Figure 4

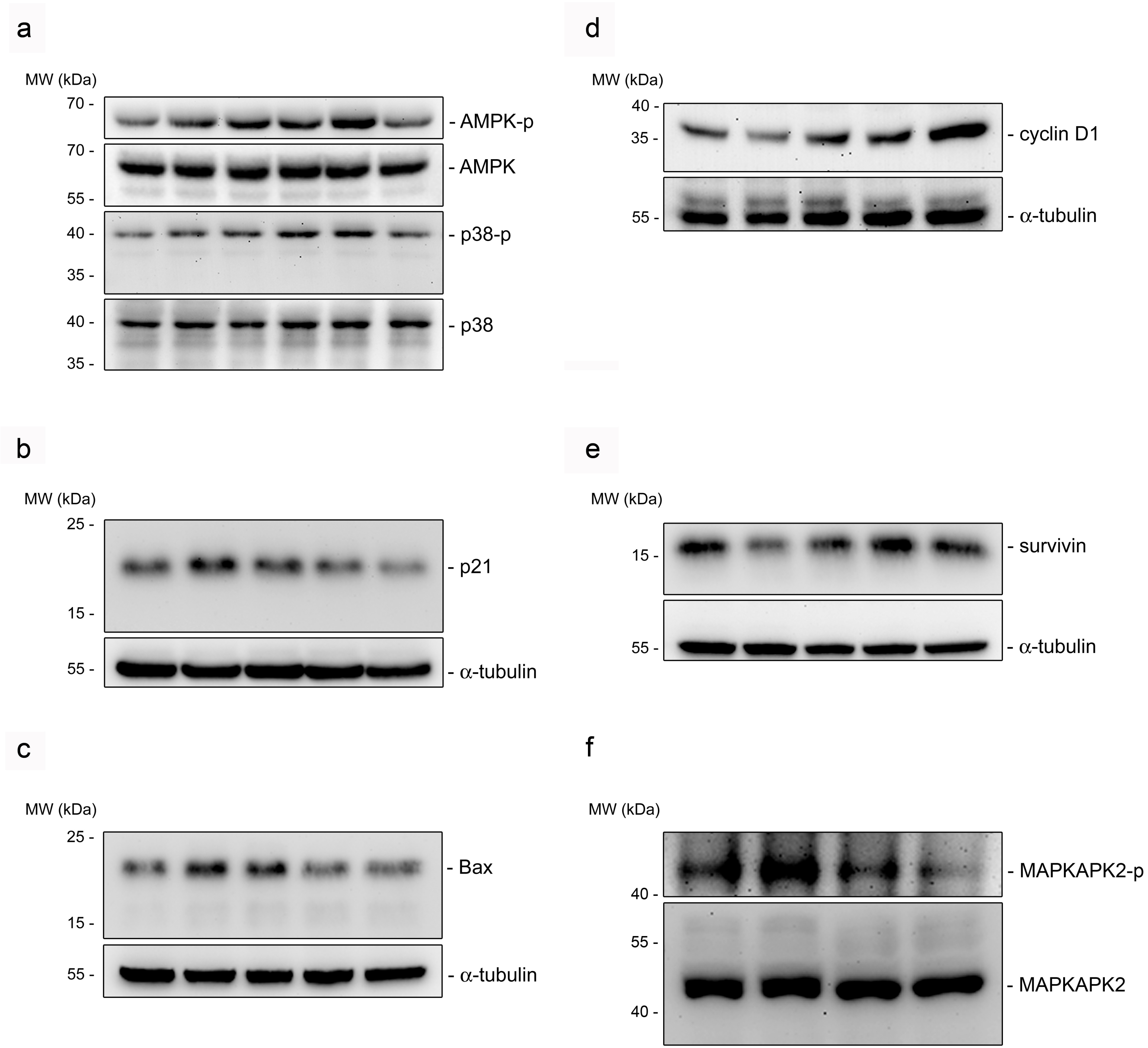

Supplementary Figure 5

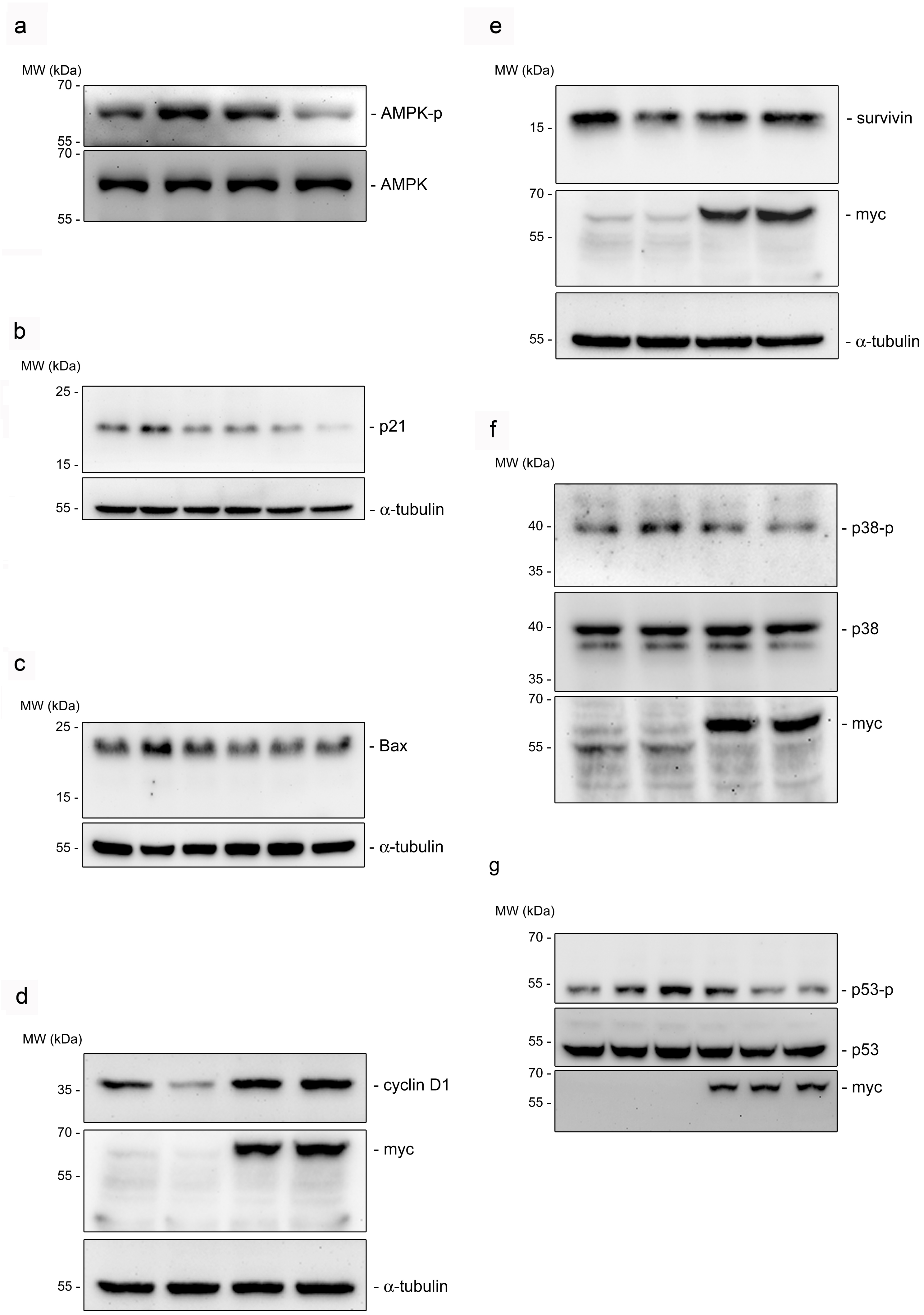

Supplementary Figure 6

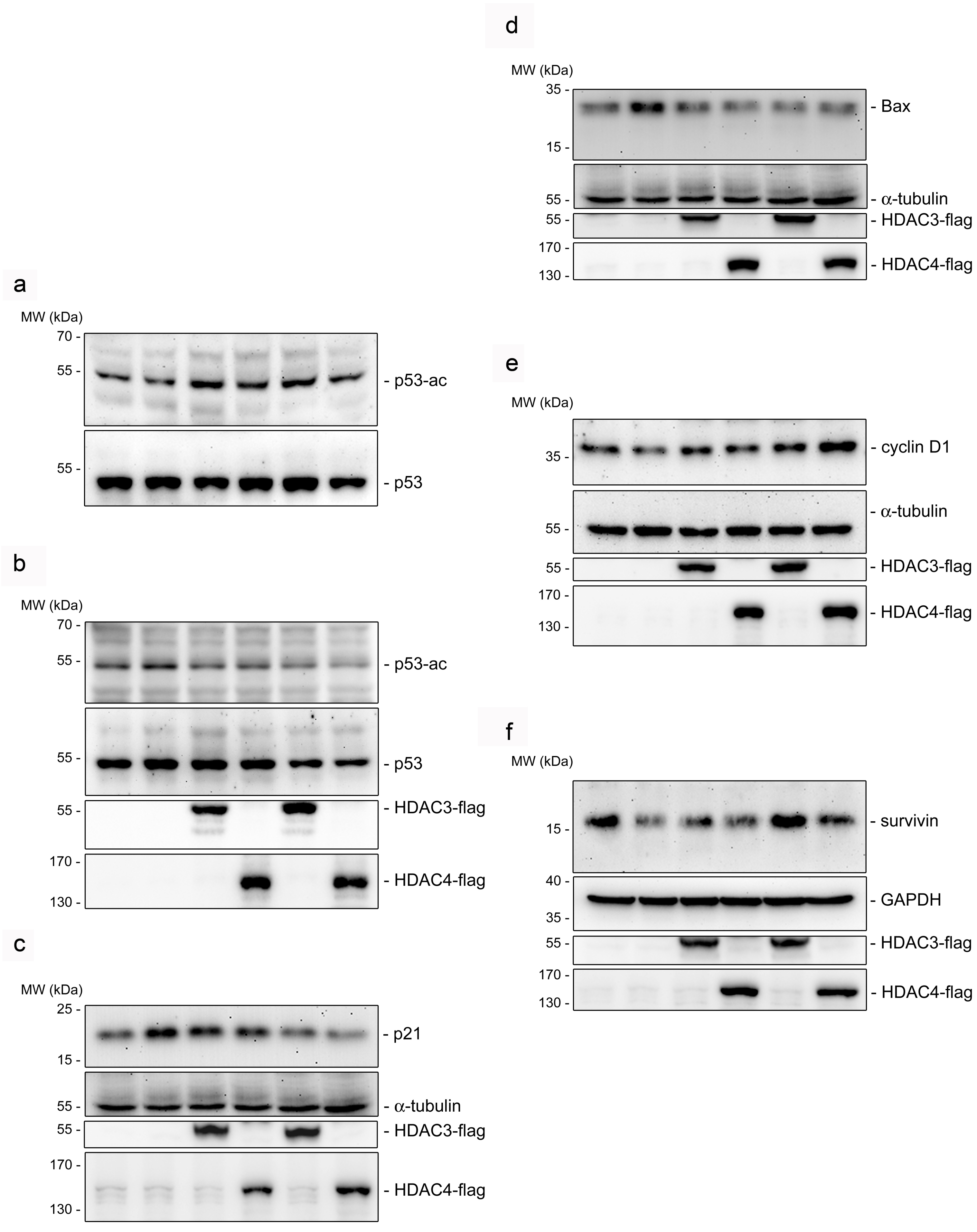

Supplementary Figure 7

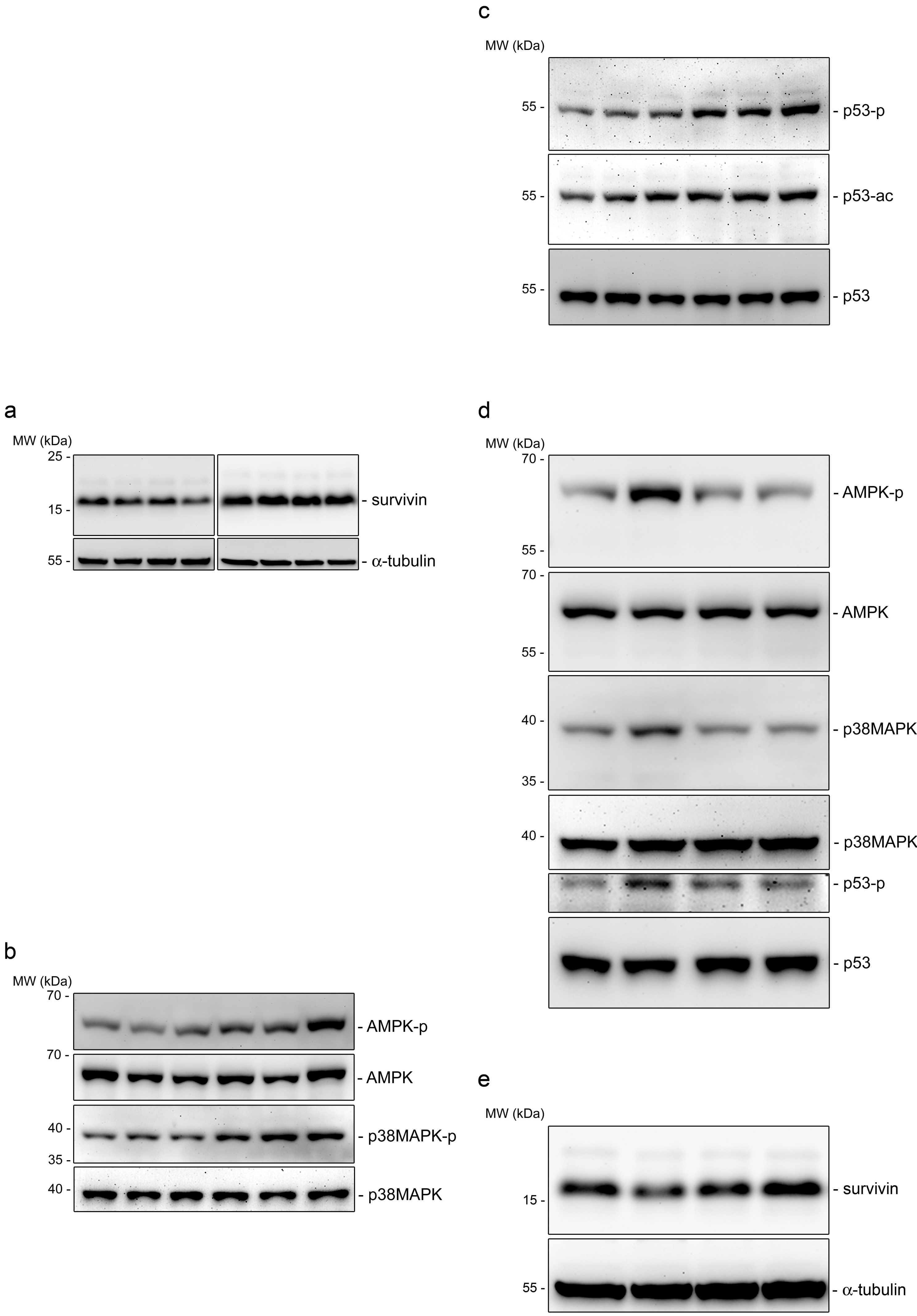

Supplementary Figure 8

a

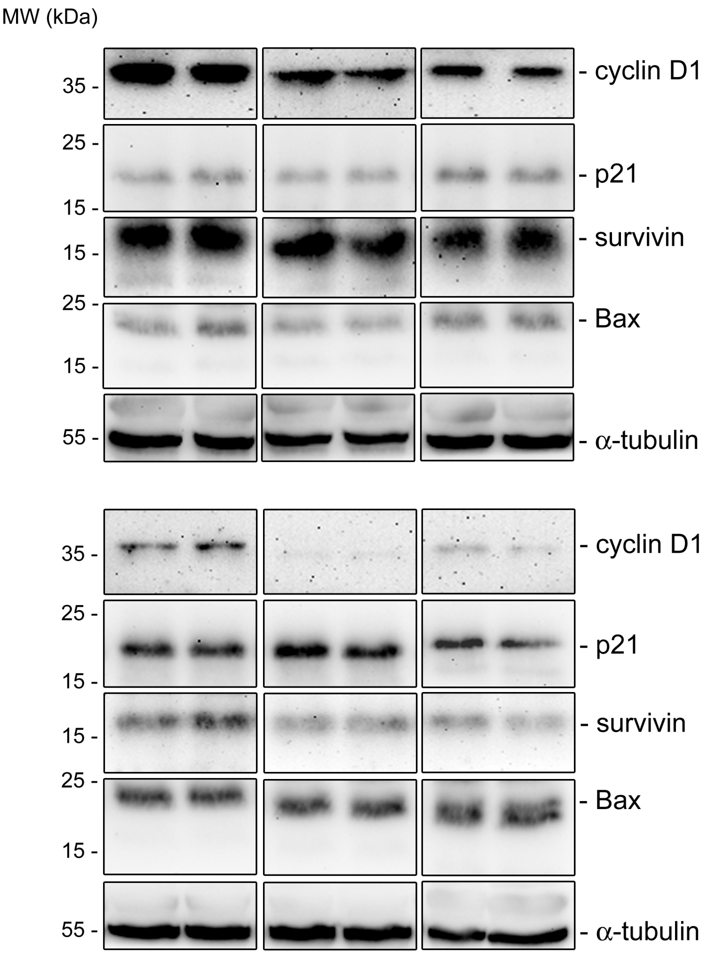

b

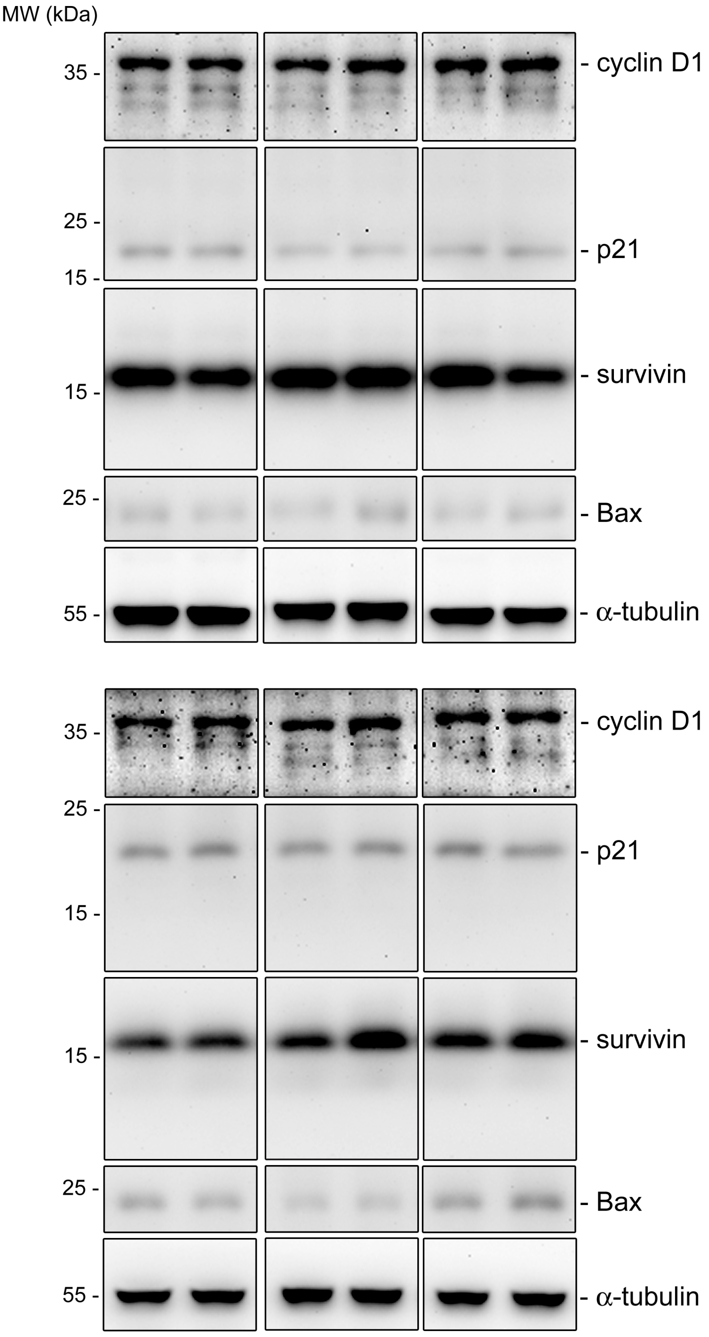

a

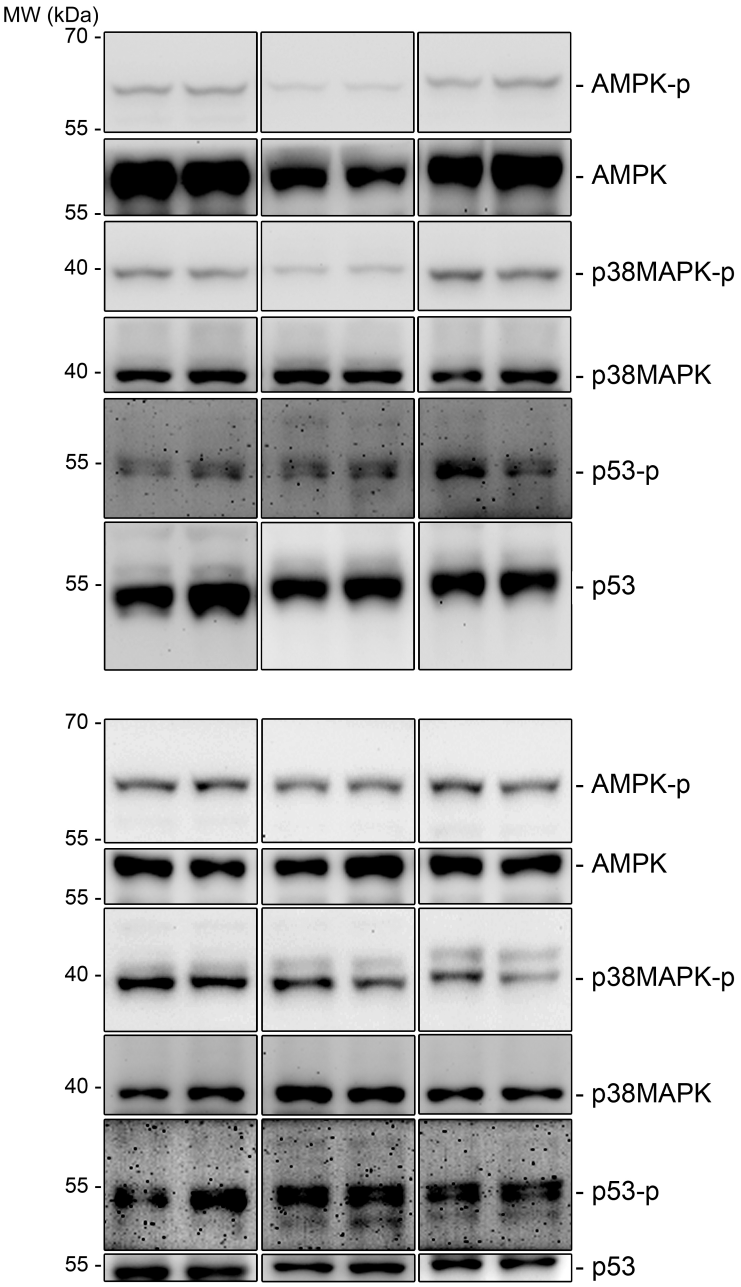
MW (kDa)
70
55
55
40
40
55
55
AMPK-p
AMPK
p38MAPK-p
p38MAPK
p53-p
p53
70
55
55
40
40
55
55
AMPK-p
AMPK
p38MAPK-p
p38MAPK
p53-p
p53
